# Gastrodin: a potential natural product for the prevention and treatment of cerebral ischemia-reperfusion injury

**DOI:** 10.3389/fphar.2025.1554170

**Published:** 2025-05-19

**Authors:** Wenxiu Qin, Jianqiang Du, Feng Wang, Junfeng Xu

**Affiliations:** ^1^ Acupuncture Department, The First Teaching Hospital of Tianjin University of Traditional Chinese Medicine, Tianjin, China; ^2^ Acupuncture Department, National Clinical Research Center for Chinese Medicine Acupuncture and Moxibustion, Tianjin, China; ^3^ Graduate School, Tianjin University of Traditional Chinese Medicine, Tianjin, China

**Keywords:** Gastrodin, cerebral ischemia-reperfusion injury, neuroprotection, pharmacological effects, traditional Chinese medicine

## Abstract

**Ethnopharmacological relevance:**

Gastrodin is the main bioactive metabolite of Gastrodia elata Blume of traditional Chinese medicine, which has pharmacological effects such as anti-inflammatory, antioxidant, neuroprotective, vasoprotective, hypoglycemic, lipotropic, analgesic, anticancer, antiviral and so on, and it has been widely used in the treatment of a wide range of diseases, especially neurological disorders.

**Aim of the review:**

Cerebral ischemia-reperfusion injury (CIRI) is defined as transient or permanent ischemia of brain tissue that is further exacerbated by restoration of blood supply. Due to the complexity of the pathological processes of CIRI, current treatments have not shown the expected effects. More and more researchers are beginning to turn their focus on combating CIRI to natural metabolites derived from botanical drugs. This review provides an overview of the progress of research on the chemical composition, pharmacokinetics, safety, and pharmacological effects of Gastrodin in the treatment of CIRI. It aims to emphasize the important pharmacological effects and mechanisms of Gastrodin in the prevention and treatment of CIRI, and to provide reference for further drug research and development, as well as the future application of Gastrodin in CIRI.

**Materials and methods:**

A systematic literature search was conducted using keywords such as “Gastrodin,” “traditional Chinese medicine,” “chemical components,” “metabolites,” “cerebral ischemia-reperfusion injury,” “CIRI,” and “pharmacological effects” to identify relevant literature published from the establishment of the database to January 2025. Databases including PubMed, Web of Science, Google Scholar, and CNKI were utilized. Raw data were included in clinical trials and animal experiments. Other studies, such as reviews and systematic evaluations, were excluded.

**Results:**

GAS can prevent and treat cerebral ischemia/reperfusion-induced neurological injury by regulating a variety of molecular signals, exerting pharmacological effects such as anti-oxidative stress, inhibition of inflammatory response, inhibition of cell death, modulation of neurotransmitters, alleviation of neurotoxicity, promotion of neural repair, protection of the blood-brain barrier, and alleviation of cerebral edema, making it a potential natural metabolite for the effective treatment of CIRI.

**Conclusion:**

Gastrodin has significant value in the treatment of CIRI and there is extensive evidence to support its use in CIRI. Further research and clinical exploration of Gastrodin is necessary to fully utilize its therapeutic potential.

## 1 Introduction

Stroke is one of the most common causes of adult disability and is considered the second leading cause of death worldwide, with ischemic stroke accounting for 70%–80% of stroke cases (Rosamond et al., 2008), making it a major threat to human life and health. Cerebral ischemia is caused by a transient or permanent reduction in blood supply, which can lead to a disruption of homeostasis in the body, including a lack of oxygen and insufficient glucose delivery (Strosznajder et al., 2010). Cerebral ischemia-reperfusion causes severe brain damage associated with complex pathological mechanisms, including mitochondrial dysfunction, release of glutamate and pro-inflammatory mediators, reactive oxygen species production, lipid peroxidation (Mehta et al., 2007), neuroexcitotoxicity and cell death during reperfusion (Gonzalez et al., 2006). Due to the complex pathology of Cerebral ischemia reperfusion injury (CIRI), current treatments still have some limitations. For example, antioxidants (e.g., edaravone) are useful for scavenging free radicals, but they are difficult to fully modulate the complex network of oxidative stress and have a short half-life, requiring frequent administration by medical personnel to maintain efficacy (Lee and Xiang, 2018). Prolonged use of anti-inflammatory drugs (e.g., glucocorticoids) may cause immunosuppression, leading to a higher risk of infection in patients and inadequate targeting of neuroinflammation (Bolshakov et al., 2021). Although neuroprotective agents (e.g., nimodipine) can improve cerebral blood flow, the vasodilatory effect of regular doses of nimodipine is limited, and high doses are prone to adverse drug reactions such as hypotension (Zhao et al., 2022), making the clinical effect more controversial. It can be seen that single-mechanism drugs are difficult to cover the complex pathologic network of CIRI, and combinations may increase toxic side effects. As a result, multi-targeted natural metabolites derived from botanical drug are gaining interest in the prevention and treatment of CIRI.

**FIGURE 1:**
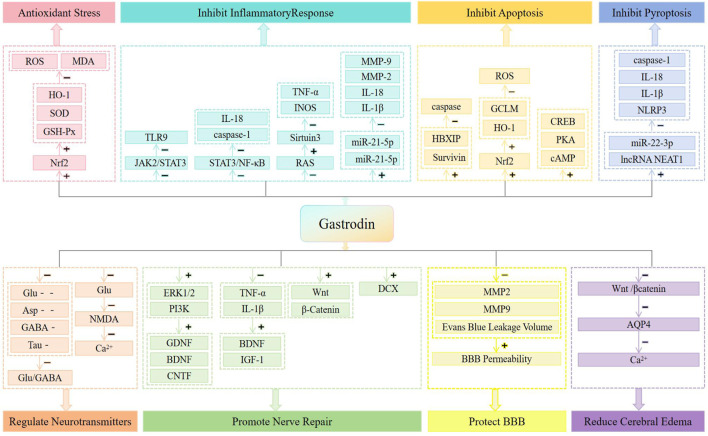
Neuroprotective mechanisms of CIRI treated with GAS.

Gastrodin (GAS), with the chemical formula C-13H18O7, is the main bioactive metabolite of Gastrodin (Liu et al., 2018a). GAS can be used for a variety of neurological disorders including cerebral ischemia-reperfusion injury, Alzheimer’s disease, Parkinson’s disease, mood disorders, cognitive disorders and neuropathic pain (Ting et al., 2021). Studies have shown that attenuation of oxidative stress and inflammatory responses and inhibition of neuronal apoptosis underlie the neuroprotective effects of GAS (Jiang et al., 2014; Zhang et al., 2018). What’s more, some animal experiments have confirmed that both preoperatively and postoperatively, GAS can improve the neurological function and reduce the infarct area and edema area in the CIRI rat model (Zeng et al., 2006), and the neuroprotective effect was even observed 7 days after reperfusion (Peng et al., 2015), which suggests that GAS has a long-lasting neuroprotective effect. In addition, compared to other natural metabolites such as Ginsenoside and Ginkgolide, GAS has neuroprotective advantages such as high bioactivity (Jensen et al., 2007), less restricted bioavailability (Atanasov et al., 2021; Sana et al., 2024; Huang et al., 2023), less toxicity (Rajaraman et al., 2006), higher safety for long-term use in high doses (Li and Li, 2022), easy passage of the blood-brain barrier (Lin et al., 2008; Wang et al., 2016), and control of blood pressure to prevent hemorrhagic transformation (Xu et al., 2024). However, we note that the potential clinical role of GAS in the treatment of CIRI has not been emphasized in recent years, and that there is a lack of a summary of the pharmacological mechanisms of GAS in the treatment of CIRI. In order to clarify the therapeutic potential of GAS and to explore its future research direction, we discussed the research progress and mechanism of GAS for CIRI in terms of pharmacology in the present work (Figure 1), with the aim of providing researchers with the most updated and useful information, drawing the attention of the general medical practitioners to GAS, providing scientific references for the development of novel multi-targeted neuroprotective strategies for the clinic and promoting the translational application of GAS in the translational application in the synergistic treatment of CIRI, and ultimately improve the prognosis of patients’ neurological function.

## 2 Pharmacokinetics and safety of GAS

GAS has high water solubility and good bioavailability, and after oral administration, GAS is rapidly converted to 4-Hydroxybenzyl alcohol (HBA) in the intestine, plasma, kidney, liver, and brain (Wang et al., 2007). β-d-glucosidase (BGL) is the key enzymes involved in the conversion of Gastrodin to p-hydroxybenzyl alcohol (HBA) (Jiang et al., 2024), this enzyme is mainly expressed in the intestine, liver and kidney. GAS has a distribution half-life in human plasma of 3.78 h and an elimination half-life of 6.06 h (Ju et al., 2010). After intravenous injection of GAS (50 mg/kg) in rats, HBA was found in bile and brain within 10 min, and peak concentrations were reached in bile and brain at 15 min, and its levels also decreased rapidly. The majority of GAS is excreted in its native form via urine, and a small proportion of GAS is reported to be excreted via bile; HBA is mainly excreted via the hepatobiliary system (Lin et al., 2007). Since GAS exerts its therapeutic effects mainly in the central nervous system, the study of GAS brain pharmacokinetics has attracted much attention. It has been reported that GAS can be detected in rats after 5 min of intravenous administration (50 mg/kg), and the peak brain concentration is reached in 15 min (Lin et al., 2008). However, the cerebral exposure of GAS to rats was small, with a cerebral blood distribution ratio of only 0.007 at a dose of 100 mg/kg administered intravenously, and it was suggested that this result may be related to the rapid metabolism of GAS to HBA (Lin et al., 2007). This implies that GAS can be rapidly distributed in the brain through the Blood-brain barrier (BBB) after entering the somatic circulation, but its very short residence time in the body and the need for multiple administrations to prolong its duration of action may limit its clinical application.

Previous studies have demonstrated that nanoparticles can enhance the penetration of drugs into the brain through the BBB (Jain et al., 2016). However, Wang’s team (Wang et al., 2025) recently proposed that most commonly used nanoparticles have trouble getting into the BBB in adult mice, and that only nanoparticles of special materials can pass through the BBB. On top of that, they found that lipid nanoparticles capable of traversing the BBB can deliver mRNA to neurons and astrocytes in a wide range of regions of the brain. Moreover, the mRNA delivery efficiency of this nanoparticle was higher than that of FDA-approved lipid nanoparticles, and it was well tolerated in multiple dosing regimens. Therefore, there is still a need to improve the preparation process of GAS-containing drugs and to explore how to enable GAS to maintain its biological concentration in the brain for a certain period of time, so as to ensure the optimal efficacy of GAS in the prevention and treatment of neurological diseases. For example, lipid nanoparticles capable of traversing the BBB are used to increase lipophilicity by surface modification and traverse the BBB using adsorption-mediated cytotransportation; or brain-derived exosomes (carrying transmembrane proteins) are used as targeting carriers to penetrate the BBB by receptor-mediated active transport. Or activate receptor-mediated BBB transport (e.g., gold nanoparticle-transferrin receptor complex) by coupling transferrin antibody, etc.; or target delivery with the help of magnetic nanoparticles combined with an external magnetic field for targeted enrichment of GAS in focal areas of the brain to improve the BBB penetration of GAS. In addition to this, slow release of GAS in the brain can be achieved by utilizing temperature-sensitive hydrogels (which form a gel after nasal administration and continuously release GAS into the cerebrospinal fluid) or microspheres/nanoparticles in long-lasting formulations. Combined with enzyme inhibitors (e.g., P-glycoprotein inhibitors) and esterase inhibitors (e.g., BNPP) to reduce the clearance of GAS by the BBB efflux pumps and premature hydrolysis in plasma, we can achieve the optimization of the metabolic stability of GAS, and synergize with other cutting-edge delivery technologies to prolong the duration of action of GAS in the brain.

Acute toxicity test in mice showed that 5.0% GAS (5000 mg/kg) was administered orally and intravenously to the tail for 3 days. No toxicity or death was observed. The subacute toxicity test in dogs and mice showed that GAS and HBA were not teratogenic or mutagenic (Lv et al., 2006). Cell experiments also showed that GAS at concentrations of 0.1–1000 µg-m L^−1^ intervened in BV-2 cells without significant toxicity (Liu et al., 2023). All these results confirm that GAS has a high safety profile.

## 3 Neuroprotective mechanisms of CIRI treated with GAS

### 3.1 Anti-oxidative stress

Oxidative stress is an important factor in the induction of brain injury during ischemia/reperfusion, during which large amounts of Reactive oxygen species (ROS) are produced, exceeding the antioxidant capacity of the brain, which activates multiple signaling pathways, leading to oxidative stress (Niizuma et al., 2009). ROS in turn initiate the expression of inflammatory cytokines, including Tumor necrosis factor-a (TNF-a), Interleukin-1β (IL-1β), and Interleukin-6 (IL-6) (Minami et al., 2006), which further exacerbate brain injury. Nuclear factor erythroid 2-related factor 2 (Nrf2) is a major regulator of several cytoprotective factors such as antioxidant enzymes, anti-inflammatory factors, and transcription factors (Ren et al., 2011; Fujita et al., 2011). Nrf2 activation plays a key role in enhancing endogenous defense mechanisms through which the brain protects itself from progressive ischemic injury (Li et al., 2013). In addition, activation of Nrf2 synergistically upregulates the expression of several antioxidant enzymes such as Heme oxygenase-1 (HO-1) and Superoxide dismutase1 (SOD1) (Cho et al., 2011; Shah et al., 2007). Thus, Nrf2 is thought to play an important role in anti oxidative stress. GAS has been reported to improve antioxidant activity in cerebral ischemic regions *in vivo*, and is able to reverse elevated Malondialdehyde (MDA) content and enhanced expression of TNF-α and IL-1β (Liu et al., 2016). GAS also increases the expression of GSH-Px, SOD and HO-1 and decreases ROS and MDA levels by activating the Nrf2 signaling pathway (Liu et al., 2020). Peng et al. (2015) also found that a high dose (100 mg/kg) of GAS increased Akt phosphorylation and Nrf2 expression, promoted SOD activity and HO-1 expression, and decreased the expression of MDA, TNF-a, and IL-1β in ischemic brain tissues of Middle Cerebral Artery Occlusion (MCAO) mice, which ultimately enhanced the neuroprotective defense mechanism through antioxidant pathway and attenuated the cerebral ischemic injury in MCAO model mice. These studies suggest that GAS can inhibit oxidative stress during CIRI.

### 3.2 Inhibiting the inflammatory response

The inflammatory response accompanies the entire course of CIRI, and inflammatory mediators, including IL-1, Inducible Nitric Oxide Synthase (INOS), and Cyclooxygenase-2 (COX-2), are upregulated following CIRI (Jin et al., 2010; Li et al., 2012). Elevated expression levels of inflammatory mediators may exacerbate neurological damage by activating various downstream pathways (Maddahi and Edvinsson, 2010). Therefore, appropriate suppression of the inflammatory response may be a potential strategy for the treatment of CIRI. NLRP3 inflammatory vesicle-induced inflammatory response plays a key role in ischemic brain injury (Minutoli et al., 2016). Zhang et al. (2019) found that GAS could inhibit the activation of NLRP3 inflammasome and attenuate CIRI-induced tissue infarction and neurological deficits by up-regulating miR-21-5p and down-regulating Thioredoxininteracting protein (TXNIP), a positive regulator of NLRP3 inflammasome, thus playing a Protective effects.

Janus-activated kinase 2 (JAK2)/Signal transducer and activator of transcription 3 (STAT3) signaling is also critical for cellular immune factor-mediated inflammatory responses (Zhu et al., 2021). Activation of JAK2 kinase by pro-inflammatory cytokines leads to rapid phosphorylation of STAT3, which migrates to the nucleus as a transcription factor and increases the expression levels of various pro-inflammatory cytokines (Huang et al., 2022). In addition, JAK2/STAT3 signaling is involved in the regulation of mitochondrial dynamics during CIRI (Zhou et al., 2019), where mitochondria are damaged during ischemia and hypoxia, affecting energy production and contributing to excess ROS production, which leads to mitochondrial DNA (mtDNA) leakage (Quan et al., 2020). Release of mtDNA during mitochondrial dysfunction can activate Toll-like receptor 9 (TLR9) signaling and initiate a pro-inflammatory response (Tripathi et al., 2023). Zhang et al. (2024) evaluated the cerebroprotective effect of GAS on CIRI by MCAO and Oxygen-Glucose Deprivation/Reperfusion (OGD/R) model based on the above research basis. The results showed that GAS could reduce infarct area, improve neurobiological function, attenuate mitochondrial damage, mtDNA leakage, and inflammatory response by inhibiting the JAK2/STAT3 signaling pathway and suppressing TLR9 expression *in vivo*. In cells, GAS rescued Oxygen glucose deprivation (OGD)-induced mitochondrial dysfunction. This reflects that the beneficial effect of GAS on CIRI is attributed to the inhibition of mitochondrial damage by GAS, which attenuates the inflammatory response. Sui et al. (2019) also found that GAS could exert anti-inflammatory effects and ameliorate astrocyte-mediated neuroinflammation during cerebral ischemia by blocking the STAT3 and Nuclear factor-kappa B (NF-κB) signaling pathways and decreasing the expression of caspase-1 and IL-18, which effectively inhibited the inflammatory vesicles in OGD-stimulated astrocytes and MCAO-responsive astrocytes.

In addition, animal experiments further demonstrated that GAS could reduce the expression of INOS and TNF-α in hypoxic-ischemic brain-damaged rats by inhibiting Renin-Angiotensin System (RAS) and activating the expression of Sirtuin3 (Liu et al., 2018b). Moreover, the combination of GAS and garcinia cambogia upregulated miR-21-5p and miR-331-5p transcription and decreased the expression of IL-1β, IL-18, Matrix metalloproteinase-2 (MMP-2), and MMP-9 in MCAO mice (Zhang et al., 2019), which attenuated CIRI-induced inflammation in the nervous system. In conclusion, these studies suggest that GAS can effectively inhibit the inflammatory response during CIRI by modulating multiple signaling pathways and reducing the expression of pro-inflammatory factors.

### 3.3 Inhibiting cells death

#### 3.3.1 Inhibition of apoptosis

Apoptosis is an important step in CIRI-induced neuronal death. Among the 12 known caspases in mammals, Cleaved caspase-3 is an important biomarker of neuronal apoptosis and the executor of apoptosis (Hartmann et al., 2000). In the MCAO rat model, GAS has been shown to maintain the expression of the anti-apoptotic protein Bcl2 and inhibit the expression of the pro-apoptotic protein Bax and CIRI-induced cleaved caspase 3 (Liu et al., 2016). Meanwhile, Survivin proteins have been shown in the literature to inhibit apoptosis (Cheng et al., 2013). Hepatitis B X-interacting protein (HBXIP), as a Survivin molecular chaperone, can form a complex with Survivin to antagonize neuronal apoptosis after CIRI (Chu et al., 2017). Xu et al. (2020) similarly found that GAS could achieve a protective effect on neurons by promoting the expression of anti-apoptotic proteins Survivin and HBXIP and inhibiting the expression of caspase apoptotic factor in a transient localized CIRI rat model. In addition, GAS activates the Nrf2 signaling pathway, increases the expression of HO-1 and Recombinant Glutamate Cysteine Ligase, Modifier Subunit (GCLM), and decreases the expression of ROS, thereby inhibiting astrocyte apoptosis in MCAO rats (Luo et al., 2018). GAS also inhibits neuronal apoptosis through activation of the cyclic Adenosine Monophosphate (cAMP)/Protein kinase A (PKA)/cAMP response element binding protein (CREB) signaling pathway (Ma et al., 2020). These studies suggest that GAS can effectively inhibit apoptosis during CIRI by regulating the expression of multiple proteins and pathways.

#### 3.3.2 Inhibition of cellular pyroptosis

Pyroptosis is a process of programmed cell death with pro-inflammatory effects distinct from apoptosis. Many studies have shown that focal death is involved in CIRI injury and plays a crucial role in CIRI induced inflammatory responses (Xia et al., 2018; An et al., 2019). Long noncoding RNAs (lncRNAs) are endogenous regulatory RNA molecules that play important roles in neurodevelopmental processes (Bao et al., 2018). Previous studies have shown that lncRNA NEAT1 promotes OGD/R-induced neuronal damage (Han and Zhou, 2019), suggesting that lncRNA NEAT1 may be involved in ischemic stroke. Zhang et al. (2021a) found that GAS could inhibit cellular focal death, attenuate CIRI induced injury in neuronal cells, improve neurological function scores in rats, and reduce the area of cerebral infarction by regulating the lncRNA NEAT1/miR-22-3p axis, down-regulating NLRP3, IL-1β, IL-18, and cleaving caspase-1. This study suggests that GAS can attenuate CIRI induced neurological injury by inhibiting cellular pyroptosis.

### 3.4 Regulation of neurotransmitters

The excitotoxicity hypothesis suggests that an imbalance between excitatory and inhibitory neurotransmitter transmission may contribute to postischemic neuroexcitotoxicity. During cerebral ischemia, neurotransmitter concentrations are altered, and excessive release of excitatory amino acids (EAA) such as Glutamic acid (Glu) or Aspartic acid (Asp) is a major pathological mechanism of cerebral ischemic injury (Tuo et al., 2022). Glu is the main excitatory neurotransmitter in the central nervous system, and in pathological conditions, EAAs have a damaging effect on nerves (Marcoli et al., 2004). The large release of EAA during cerebral ischemia is accompanied by a large release of Inhibitory amino acids (IAA), such as γ-aminobutyric acid (GABA) and Taurine (Tau), which can somewhat counteract the toxicity of EAA (Bogaert et al., 2000).

Bie et al. (2007) analyzed the true time-dynamic process of amino acid changes in the brain of CIRI rats by establishing a CIRI model and applying brain microdialysis combined with high-performance liquid chromatography (HPLC) technology, and found that GAS significantly inhibited the elevation of the levels of four amino acids, namely, Glu, Asp, GABA, and Tau in the striatum during the process of I/R, and the inhibitory effect on EAA was more significant than that on IAA, and it was also found that GAS clearly reduced the elevated Glu/GABA ratio after ischemia, thus reducing the volume of cerebral infarction in cerebral I/R rats. This indicates that the neuroprotective mechanism of GAS after CIRI may be related to its regulation of neurotransmitter homeostasis. Zeng et al. (2007) also found that in the early stage of CIRI, pre-ischemic administration of GAS obviously reduced elevated Glu levels after cerebral ischemia, increased extracellular GABA content during reperfusion, decreased the Glu/GABA ratio during cerebral ischemia/reperfusion, and regulated imbalanced neurotransmitters after cerebral I/R, which then ameliorated Cerebral Ischemia/Reperfusion (I/R)-induced excitotoxicity. Meanwhile, during neuronal ischemia and hypoxia, cytoplasmic Ca^2+^ overload triggers neuronal degeneration when N-methyl-D-aspartic acid receptor (NMDA) is stimulated by excess Glu (Lee et al., 1999). Their team’s *in vitro* experiments also found that GAS obviously inhibited OGD-induced increases in Ca^2+^ and Nitric Oxide (NO), and reduced extracellular Glu levels and neuronal cell death after OGD (Zeng et al., 2006). Xu et al. (2007) found that 24 h of hypoxia could cause significant neuronal damage, while the addition of GAS pretreatment could inhibit the hypoxia-induced increase in extracellular Glu levels and reduce the level of cytoplasmic overloaded Ca^2+^ during NMDA injury, resulting in the prevention of hypoxia-induced neurotoxicity and a substantial increase in neuronal survival rate. This study implies that the application of GAS prior to an ischemic event may also modulate neurotransmitter homeostasis and combat CIRI. In summary, GAS ameliorates CIRI-induced neurological injury by modulating neurotransmitter homeostasis and attenuating neurotoxic substance release.The neuroprotective effects of GAS modulation of specific neurotransmitters in CIRI are shown in Table 1.

**TABLE 1:** GAS modulates the neuroprotective effects of specific neurotransmitters in CIRI.

Model	Animal/Cell	Neurotransmitter^a^	Measurement Methods	Ref.
MCAO/R	Male SD rats	↓↓Glu; ↓↓Asp; ↓GABA; ↓Tau; ↓Glu/GABA	Cerebral microdialysis-HPLC technology	Bie et al. (2007)
MCAO/R	Male SD rats	↓Glu; ↑GABA; ↓Glu/GABA	High-performance liquid chromatography (HPLC)	Zeng et al. (2007)
OGD	Primary hippocampal cell	↓Glu	HPLC	Zeng et al. (2006)

^a^ ↓, decrease; ↑, improve.

### 3.5 Promoting nerve repair

Neurotrophic factors are proteins essential for neuronal growth and survival; therefore, the neurotrophic effects of GAS are expected to promote neuronal repair. The current study suggests that GAS can promote neurological recovery by promoting the secretion of Brainderived neurotrophic factor (BDNF) in injured neural tissues (Song et al., 2013). The proliferation and migration of Schwann cells play a crucial role in the process of neural repair. GAS can promote the expression of Glial cell line-derived neurotrophic factor (GDNF), BDNF, and Ciliary neurotrophic factor (CNTF) by activating the Extracellular regulated protein kinases 1/2 (ERK1/2) and Phosphoinositide 3-kinase (PI3K) signaling pathways, and induce the proliferation of RSC96 Schwann cells. Line-derived neurotrophic factor (GDNF), BDNF and Ciliary neurotrophic factor (CNTF), and induced the proliferation of RSC96 cells (Zuo et al., 2016). It has also been shown that GAS can enhance the cellular activity and migration function of OGD-induced astrocytes by inhibiting the expression of TNF-α and IL-1β, promoting the expression of BDNF and Insulin-like growth factor I (IGF-1) (Zhang et al., 2021b), and promoting neuronal recovery.

In addition to neurotrophic factors, Doublecortin (DCX), a microtubule-associated protein, is also involved in the migration and differentiation of newborn neurons, and the amount of DCX can indirectly reflect the strength of hippocampal neurogenesis (Schloesser et al., 2015). Animal studies revealed that MCAO/R caused downregulation of hippocampal DCX expression in mice after MCAO/R, whereas a certain dose of GAS could upregulate the expression level of hippocampal DCX, which exerted a neuroprotective effect on the migration and differentiation of newborn neurons (He et al., 2015). This shows that GAS may regulate hippocampal neurogenesis by up-regulating the expression level of hippocampal DCX, which in turn plays a neuroprotective role during CIRI. During cerebral ischemia/reperfusion, not only neurotrophic factors and microtubule-associated proteins are involved in promoting neural repair, but some signaling pathways are also closely related to the promotion of neural repair, and the Wnt/β-Catenin signaling pathway is one of them. The Wnt signaling pathway is part of an evolutionarily conserved intracellular signal transduction cascade that regulates several critically important processes such as cell proliferation, differentiation, and migration (Eubelen et al., 2018; Alves and Smidt, 2011). The Wnt/β-Catenin signaling pathway is the binding of Wnt protein ligands to the receptors of Frizzled (FZD) and low-density lipoprotein receptor-related proteins 5 and 6 (LRP5/6) binding. It activates a series of complex biochemical reactions that block the cytoplasmic Wnt/β-Catenin degradation pathway, resulting in the accumulation of β-catenin in the cytoplasm, followed by its translocation to the nucleus, where it assembles with T-cell factors (TCF/LEF) to form a transcription complex (Routledge and Scholpp, 2019), regulates neurogenic protein expression and promotes neurogenesis (Belinson et al., 2016).

Previous studies have shown that Wnt signaling promotes functional recovery after focal ischemic injury by increasing neurogenesis (Shruster et al., 2012), and plays an important regulatory role in maintaining cerebrovascular and neuronal cell functions (Menet et al., 2020). Several studies have also reported that Wnt/β-Catenin signaling is an endogenous protective mechanism in the adult brain and that activation modulates CNS diseases (Cheng et al., 2022). Interestingly, glycogen synthase kinase 3β (GSK-3β) normally activates β-Catenin in the absence of Wnt signaling, leading to its degradation by proteases. When Wnt signaling is activated, GSK-3β activity is inhibited, and β-Catenin is able to accumulate and enter the nucleus to play a role, thus favoring neuronal survival (Tang et al., 2010). Qiu et al. (2019) used Western blotting to detect the expression of key proteins in the Wnt/β-Catenin signaling pathway affecting neuroprotection and neurogenesis in MCAO mice. The results showed that the expression of Wnt 3a and β-Catenin would be decreased after MCAO, whereas administration of medium-dose GAS (100 mg/kg) upregulated the expression of Wnt 3a and β-Catenin. They also assessed the expression of functional proteins downstream of the Wnt/β-Catenin signaling pathway and found that GAS regulated the expression of Ngn2, Tbr2, and Pax6 in ischemic cortex. Ngn2, Tbr2, and Pax6 have been shown to be important neurogenesis promoters in the Wnt/β-Catenin signaling pathway. Ngn2 promotes hippocampal neuroblast differentiation (Roybon et al., 2009). Tbr2 promotes the transition of radial glial cells to intermediate progenitors (Englund et al., 2005). Pax6 induces differentiation of olfactory bulb interneurons (Imamura and Greer, 2013). In conclusion, it was experimentally confirmed that GAS could protect the Wnt/β-Catenin signaling pathway and regulate the downstream expression of Ngn2, Tbr2, and Pax6 in MCAO mice, thereby promoting neurogenesis, reducing infarct volume, and improving neurological function in mice after MCAO. This suggests that the protective effect of GAS on neurons after CIRI may be achieved by modulating the Wnt/β-Catenin signaling pathway.

### 3.6 Protecting the blood-brain barrier

An important adverse consequence of CIRI is BBB disruption (Molina and Alvarez-Sabin, 2009). The BBB is a complex dynamic structure that acts as a barrier between blood and brain tissue, preventing brain damage and controlling molecular and ionic exchange between brain and blood (Theodorakis et al., 2017). If this exchange is disrupted, it will result in a compositional imbalance between blood and brain. Ischemia and hypoxia induce disruption of the integrity of connective proteins in brain endothelial cells, such as occludin and claudin proteins, leading to increased BBB permeability (Abdullahi et al., 2018). However, increased BBB permeability causes BBB dysfunction, which fails to control the exchange of substances between the peripheral circulation and the CNS, disrupting the homeostasis of CNS tissues, a major consequence of hemorrhagic transformation and CIRI. Li et al. (2019) found that GAS pretreatment reduced Evans Blue leakage volume, decreased MMP2 and MMP9 expression, reversed BBB injury, attenuated inflammation, reduced the area of cerebral infarction, and improved neurobehavioral deficits in CIRI rats. In summary, their study suggests that GAS preconditioning helps to restore connexin expression and BBB repair after ischemia/reperfusion injury, which facilitates the recovery of CIRI.

### 3.7 Reducing cerebral edema

Currently, the role of Aquaporins (AQPs) in the occurrence and development of cerebral edema is receiving increasing attention, in which AQP4, a water channel protein controlling the entry and exit of water molecules into and out of the cell membrane, has a very important role in the maintenance of water homeostasis of brain tissues, inflammation, and regulation of neural excitability (Miu and Li, 2011). When cerebral ischemia occurs, the regulation of AQP4 causes changes in cell membrane permeability (Papadopoulos and Verkman, 2007), which in turn activates the Transient receptor potential vanilloid receptor 4 (TRPV4) channel, prompting Ca^2+^ to enter the cell smoothly through the channel, and when the osmotic pressure difference between inside and outside of the cell is generated water enters the cell, which this ultimately leads to swelling of nerve cells (Tureckova et al., 2023). It was found that GAS could inhibit the expression of AQP4 in the cortical ischemic semi-dark zone region of CIRI rats through negative regulation of the Wnt/βcatenin signaling pathway, reduce the entry of Ca^2+^ into the cells, and attenuate cerebral edema, which in turn protects the neuronal cells and improves the impairment of neurological function of the rats, with a dose-dependency (Xie et al., 2022). This study demonstrated that GAS helps to reduce cerebral edema, thereby ameliorating neurological damage during cerebral ischemia/reperfusion. Animal experiments of GAS treatment for CIRI are shown in Table 2.

**TABLE 2:** Animal experiments on GAS treatment of CIRI.

Model	Animal	Dosage of gastrodin^a^	Major findings	Ref.
MCAO/R	C57BL/6J mice	Low-dose, L = 10 mg/kg, i.p. Medium-dose, M = 50 mg/kg, i.p. High-dose, H = 100 mg/kg, i.p.	High dose GAS (100 mg/kg) reduces tested neuronal injury and neurobehavioral deficient	Peng et al. (2015)
MCAO/R	Male C57BL/6 mice	100 mg/kg, i.p.	GAS suppresses inflammasome activation and exerts a protective effect by regulating the miR-21-5p target TXNIP	Zhang et al. (2019)
MCAO/R	Male SD rats	100 mg/kg, i.p.	GAS therapies for cerebral ischemia by inhibiting JAK2/STAT3 signaling	Zhang et al. (2024)
MCAO/R	Male SD rats	Low-dose, L = 15 mg/kg, i.p. Medium-dose, M = 30 mg/kg, i.p. High-dose, H = 60 mg/kg, i.p.	GAS ameliorates subacute phase cerebral I/R injury by inhibiting inflammation and apoptosis (differences in concentration were not emphasized)	Liu et al. (2016)
MCAO/R	Male SD rats	Low-dose, L = 25 mg/kg, i.p. Medium-dose, M = 50 mg/kg, i.p. High-dose, H = 100 mg/kg, i.p.	GAS improves ischemic stroke by promoting anti-apoptotic protein expression and inhibiting caspase-mediated neuronal death Gas (50, 100 mg/kg) is more effective than Gas (25 mg/kg)	Xu et al. (2020)
MCAO/R	Male SD rats	50 mg/kg, i.p.	GAS reduces neuronal cell pyroptosis by decreasing the levels of NLRP3 and cleaved caspase-1	Zhang et al. (2021a)
MCAO/R	Male SD rats	50 mg/kg, i.p.	GAS decreases the volume of cerebral infarction, ameliorates the cerebral injury by improving the level of amino acids in striatum	Bie et al. (2007)
MCAO/R	Male SD rats	100 mg/kg, i.p.	Administration of GAS before ischemia significantly reduced the ischemia-induced elevation of glutamate levels during the postischemic period, increased the rise of extracellular GABA during the reperfusion periods, thus decreased the glutamate/GABA ratios during CIRI	Zeng et al. (2007)
MCAO/R	Male C57BL/6 J mice	Low-dose, L = 10 mg/kg, i.p. Medium-dose, M = 50 mg/kg, i.p. High-dose, H = 100 mg/kg, i.p.	GAS may regulate hippocampal neurogenesis by upregulating the expression level of hippocampal DCX, and then exert neuroprotective effect on CIRI Gas (50, 100 mg/kg) is more effective than Gas (10 mg/kg)	He et al. (2015)
MCAO/R	Male SD rats	Low-dose, L = 50 mg/kg, i.p. Medium-dose, M = 100 mg/kg, i.p. High-dose, H = 200 mg/kg, i.p.	Evans blue leakage volume was reduced with GAS pretreatment notably at dose 100 mg/kg	Li et al. (2019)
MCAO/R	Male SD rats	Low-dose, L = 15 mg/kg, i.g. Medium-dose, M = 30 mg/kg, i.g. High-dose, H = 60 mg/kg, i.g.	GAS reduces neurological deficits, inhibits cerebral edema, and reduces neuronal cell damage in a dose-dependent manner	Xie et al. (2022)

^a^ i.p., intraperitoneal administration; i.g., intragastrical administration.

## 4 Conclusion and future perspectives

GAS has been broadly used in clinical practice for the treatment of central nervous system disorders such as convulsive disorders, headaches, dizziness, stroke, epilepsy, and amnesia. Nevertheless, GAS has not been well studied in the prevention and treatment of CIRI, and a comprehensive understanding of the potential mechanisms and targets of GAS in the prevention and treatment of CIRI is essential for the discovery and design of effective therapeutic strategies. From the above numerous animal experiments, it can be found that GAS can prevent and treat cerebral ischemia/reperfusion-induced neurological injury by regulating a variety of molecular signals, exerting pharmacological effects such as anti-oxidative stress, inhibition of inflammatory response, inhibition of cell death, modulation of neurotransmitters, alleviation of neurotoxicity, promotion of neural repair, protection of the blood-brain barrier, and alleviation of cerebral edema, making it a potential natural metabolite for the effective treatment of CIRI. However, the following questions remain about current research on GAS for the prevention and treatment of CIRI.

First, a systematic search of a large body of literature revealed that current pharmacological studies of GAS for CIRI mainly focus on animal experiments, with significant research paradigm limitations. Moreover, Some of the animal experiments lacked a description of the purity of the GAS, or the purity used lacked a reference, and the design protocols were not sufficiently rigorous. At the same time, the research phase is overly focused on exploring the mechanism of neuroprotective effects in the acute phase, and generally lacks a long-term follow-up observation design, which has yet to provide conclusive evidence to support the potential value of this therapy in the treatment of CIRI in terms of long-term prognosis. In addition to this, animal experiments usually compare different dose gradients (low, medium, high) of GAS longitudinally with sham-operated/modeled groups, and there is a lack of cross-sectional efficacy comparisons with current clinically used neuroprotective agents (e.g., edaravone, nimodipine), and little research on synergistic effects with novel interventions (e.g., hypothermia, stem cell therapy). This one-dimensional experimental design has resulted in the current inability to establish an efficacy hierarchy between GAS and other therapeutic options through evidence-based medicine, and the lack of systematic documentation of key clinical symptoms and quantitative analysis of the duration of interventions for GAS treatment of CIRI. Overall, the scientific quality of the original studies related to GAS treatment of CIRI was low (The assessment of the scientific quality of the original study is shown in Table 3). Therefore, for the time being, it cannot be shown that GAS has better efficacy than other interventions. It is worth noting that many of the animal experiments mentioned above have shown that GAS can indeed treat CIRI-induced neurological injury through multiple mechanisms and pathways, and is a more promising drug for combating CIRI. Based on the above issues, it is suggested that a four-tier validation system should be established for future research: (i) Conducting randomized controlled trials of GAS with positive control drugs; (ii) Constructing a mechanism validation platform for multi-omics joint analysis; (iii) Designing multi-center clinical trials to assess the clinical translational value; (iv) Tracking the long-term efficacy of GAS in the treatment of CIRI. This multidimensional research strategy will help clarify the unique position of GAS in the therapeutic spectrum of CIRI and provide theoretical support for the development and translation of novel neuroprotective agents.

**TABLE 3:** Assessment of scientific quality of original research.

Materials and sources	Model Preparation	GAS purity^a^	GAS Dosage reference	Research Reliability	Experimental design	Ref.
Detailed and clear	standardize	99.2%	Yes	Very reliable	Very reasonable	Peng et al. (2015)
Detailed and clear	standardize	-	No	General	General	Zhang et al. (2019)
Detailed and clear	standardize	>98%	No	Relatively reliable	Reasonable	Zhang et al. (2024)
Detailed and clear	standardize	>98%	No	Relatively reliable	Reasonable	Liu et al. (2016)
Detailed and clear	standardize	-	No	General	General	Xu et al. (2020)
Detailed and clear	standardize	98%	No	Relatively reliable	Reasonable	Zhang et al. (2021a)
Detailed and clear	standardize	99.6%	No	Relatively reliable	Reasonable	Zeng et al. (2007)
Detailed and clear	standardize	-	No	General	General	He et al. (2015)
Detailed and clear	standardize	-	No	General	General	Li et al. (2019)
Detailed and clear	standardize	-	No	General	General	Xie et al. (2022)

^a^ -: Not mentioned in the original document.

Second, a critical analysis of the current body of evidence reveals that studies on the pharmacodynamic mechanisms of GAS in the prevention and treatment of CIRI exhibit significant multidimensional features. None of the available studies can support the hypothesis of a single mechanism of GAS against CIRI (e.g., independent antioxidant, anti-inflammatory, or anti-apoptotic pathways), but rather, neuroprotective effects are realized through multimodal regulatory networks. Although the research teams have the phenomenon of selective focusing of mechanisms due to experimental design: for example, some studies focused on the role of anti-inflammatory mechanism of GAS in CIRI, while others focused on the role of anti-apoptotic mechanism of GAS in CIRI, etc., their results showed that there is cross synergy between these mechanisms. However, the results of their studies all indicate that there is cross-synergy between these mechanisms, which suggests that the prevention and treatment of CIRI by GAS is the result of the joint action of multiple mechanisms, pathways and targets. Notably, even so, future studies should be analyzed in a multi-omics integration to reveal the most central regulatory hubs of GAS for the prevention and treatment of CIRI and to explore the unique advantages of GAS for the prevention and treatment of CIRI.

Third, there are fewer studies related to the neuroprotective effects of GAS-modulated neurotransmitters in CIRI, and most of the existing studies are limited to a single transmitter class (e.g., glutamate or GABA), and there is a tendency towards technological homogenization at the level of the assay methodology (which relies mainly on HPLC technology). In light of this, future studies should also further explore the expression of other neurotransmitters (e.g., dopamine, 5-HT, ACh, Adenosine, and BDNF, among others) in CIRI and how the GAS exerts neuroprotective effects by modulating these neurotransmitters. It also complements and refines future quantitative neurotransmitter measurement techniques through the following dimensions: (i) Real-time dynamic monitoring, utilizing microdialysis and electrochemical sensing techniques, using implantable microprobes combined with HPLC or mass spectrometry to detect relevant neurotransmitters in the extracellular fluid. (ii) Utilize molecular imaging techniques to quantify the occupancy or release pattern of neurotransmitter receptors. (iii) Rationalize the use of metabolomics and single-cell analysis techniques to localize the distribution of neurotransmitters in the brain by spatial metabolomics. And use single-cell sequencing technology to identify the expression of transmitter synthase in specific neuronal subtypes. (iv) Dynamic monitoring of neurotransmitter fluorescently labeled promoters by gene and protein expression analysis. And quantify transmitter transporters (e.g., VGluT1, VGAT, etc.) in synaptosomes by Western blot/ELISA. (v) Construct a neuroprotective effect prediction framework by integrating transmitter concentration, imaging data and behavioral results through machine learning. In conclusion, future studies will need to incorporate novel probes, artificial intelligence analysis and interdisciplinary approaches to precisely resolve the protective mechanisms of neurotransmitter networks and drive drug development for individualized neuroprotective therapies.

Finally, so far, animal experiments and clinical trials have not investigated the minimum active concentration and the dose-effect relationship of GAS for the treatment of CIRI, and therefore, we are not able to clarify the minimum active concentration and the optimal dosing time of GAS for the treatment of CIRI. In addition, compared with a large number of basic experiments, there is a lack of clinical trials related to the prevention and treatment of CIRI with GAS, and the methodological quality of the existing clinical studies is generally low. Therefore, there is an urgent need for more robust clinical trials to confirm the therapeutic activity of GAS in humans and further studies are still needed to elucidate in detail the cerebral pharmacokinetics, preparation standardization, and optimal human therapeutic dose of GAS. Furthermore, in terms of future translational applications of GAS, in addition to the GAS nano-delivery regimen proposed in the pharmacokinetics section, cryotherapy and stem cell therapy are both therapies that have been shown to have neuroprotective effects (Yang et al., 2025). It is expected that in the future, researchers will develop GAS nano-retarded release formulations for use in conjunction with cryogenic devices for emergency or intensive care in CIRI. It is also expected that they can construct GAS-stem cell co-delivery systems (e.g., hydrogel carriers, etc.) for nerve regeneration after CIRI. And we will conduct a multicenter RCT study to explore the dose-effect relationship and long-term safety issues of GAS combination therapy, etc. In summary, GAS should be considered as a multi-target drug for the treatment of CIRI to achieve more research and promotion in the future.
